# 3D Printed Patient-Specific Cutting Guides for Bone Grafting in Reverse Shoulder Arthroplasty: A Novel Technique

**DOI:** 10.1177/24715492231162285

**Published:** 2023-03-14

**Authors:** Jillian N Karpyshyn, Aaron J Bois, Heather Logan, Graeme T Harding, Martin J Bouliane

**Affiliations:** 1 Department of Orthopaedic Surgery, 3158 University of Alberta, Canada; 2 Section of Orthopaedic Surgery, Department of Surgery, Cumming School of Medicine, 2129 University of Calgary, Calgary, AB, Canada; 3 McCaig Institute for Bone and Joint Health, 2129 University of Calgary, Calgary, AB, Canada; 4 Institute for Reconstructive Sciences in Medicine, 3158 University of Alberta, Canada

**Keywords:** glenoid bone loss, reverse shoulder arthroplasty, patient-specific cutting guides

## Abstract

Glenoid bone loss remains a challenge in shoulder arthroplasty. Addressing substantial bone loss is essential to ensure proper function and stability of the shoulder prosthesis and to prevent baseplate loosening and subsequent revision surgery. Current options for creating and shaping glenoid bone grafts include free-hand techniques and simple reusable cutting guides that cut the graft at a standard angle. There is currently no patient-specific device available that enables surgeons to accurately prepare the bone graft and correct glenoid deformity. We present a novel surgical technique using three-dimensional (3D)-printed cutting guides to create a patient-specific bone graft to address glenoid deformity in the setting of reverse shoulder arthroplasty.

## Introduction

Managing patients with substantial glenoid bone loss secondary to arthritis, cuff tear arthropathy or dysplasia remains a challenge in shoulder arthroplasty.^1,2,3^ In the setting of reverse shoulder arthroplasty (RSA), insufficient glenoid bone stock threatens proper positioning and fixation of the glenoid baseplate and increases the risk of inferior scapular notching and perimeter impingement, premature glenoid component loosening and/or failure, and instability.^4,7^ Restoration of the joint line optimizes deltoid tension and ‘wrapping’ of the deltoid which is crucial for shoulder function and stability.^5,6^

Boileau et al^8^ recently described a technique for the use of angled bony-increased offset RSA (angled BIO-RSA) to address multiplanar glenoid deformity. They demonstrated that the use of an angled bone graft resulted in a reliable and predictable union of the graft with the native glenoid, correction of malalignment, and successful lateralization of the glenoid component. This technique has demonstrated good results in the literature with high rates of graft incorporation and decreased complications.^9,10,11,12^ Such procedures require minimal access to specialized equipment but are technically challenging and time-consuming and fail to address the variability in graft size/shape that is required to address glenoid deformities between patients.^9,10,11^ While there are simple reusable cutting guides available to prepare either a symmetrical or angled bone graft, there is currently no patient-specific device available that enables surgeons to accurately prepare the bone graft and correct glenoid deformity.

The use of patient-specific instrumentation (PSI), surgical design and simulation (SDS) and three-dimensional (3D) printing has rapidly evolved in medicine and its use is expanding in certain areas of orthopedic surgery, including but not limited to, orthopedic oncology, arthroplasty, and deformity correction surgery.^13,14,15,16^ PSI involves creating surgical instruments based on a patient's unique anatomy and using this technology to implant components in an optimal position. This is particularly relevant in cases with severe glenoid deformity or deficient bone stock which increases the risk for suboptimal glenoid component positioning and fixation. SDS and 3D-printed cutting guides are commonly used in other surgical areas that require bone graft reconstruction such as mandibular reconstruction and bone tumor surgery.^17,18,13,14^ Current options for creating and shaping glenoid bone grafts are limited and include free-hand cutting (ie, no surgical guide used) or the use of a reusable cutting guide that cuts the graft at a standard size and angle.

At our institution, when confronted with substantial glenoid deformity, surgeons have also used SDS and a 3D-print of the patient's scapula and humerus to create a custom (ie, patient-specific) single-use 3D-printed cutting guide. This permits efficient and accurate preparation of the bone graft, utilizing humeral head autograft, to the precise dimensions required to correct each patient's glenoid deformity (ie, individualized to the patient's deformity). The purpose of this study was to present a novel surgical technique utilizing a patient-specific 3D-printed cutting guide to create a customized bone graft to precisely match the glenoid deformity in the setting of RSA with glenoid bone loss.

## Surgical Technique

### Patient Evaluation

The patient is assessed in the clinic setting and a standard history is obtained and a physical examination is performed taking note of shoulder strength, range of motion, and the presence of lag signs. Plain radiographs consisting of true anteroposterior and axillary views of the shoulder are obtained. Preoperatively, informed consent was obtained from each patient for the publication of the case details and accompanying imaging.

### Preoperative Planning

**Figure 1.**
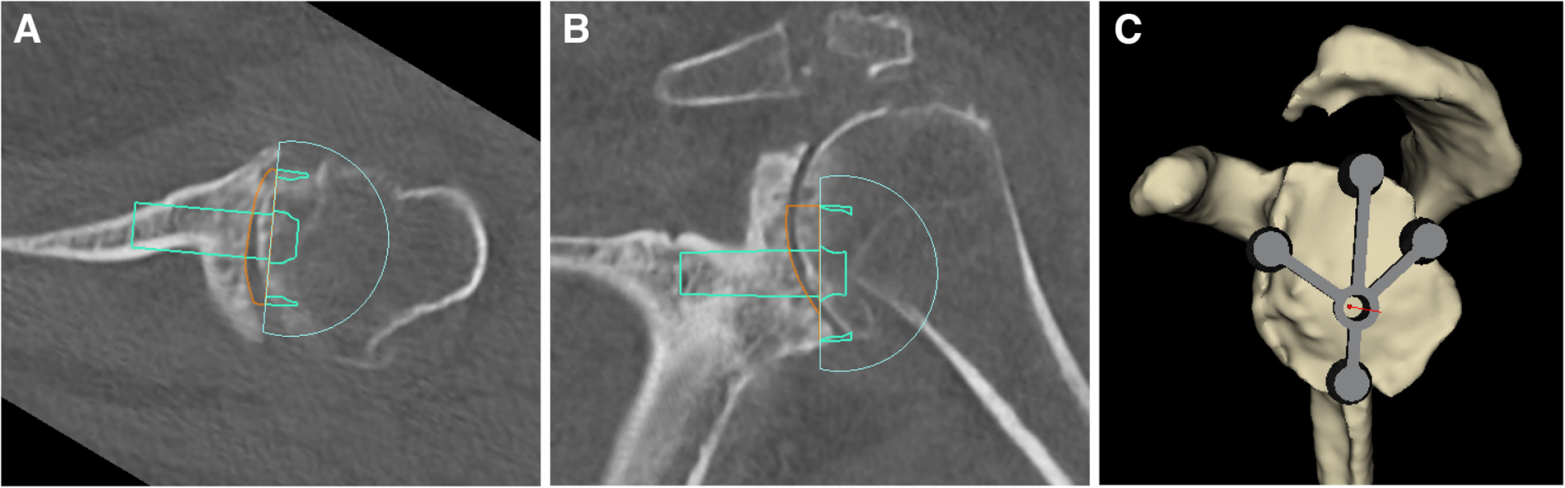
(A & B) three-dimensional (3D) planning software is used to determine optimal position of the glenoid baseplate to correct the glenoid deformity. (C) The planned location and orientation of the glenoid guide pin is templated.

A high-resolution computed tomographic (CT) scan of the operative shoulder is obtained to assess glenoid version and inclination (ie, glenoid deformity). Images are exported in Digital Imaging and Communications in Medicine (DICOM) format to facilitate planning and 3D-printing of the cutting guides. Preoperative planning is performed with 3D planning software (BLUEPRINT™, Wright Medical) which permits accurate and reproducible measurements of version/inclination and the graft dimensions required to address the glenoid deformity^19,20,21^ (Figure 1). The virtual model is then used to template the planned location and orientation of the glenoid guide pin. The “patient-specific bone graft” option is selected once the glenoid baseplate has been placed in the desired position that optimizes deformity correction. The patient-specific bone graft is then evaluated for its shape (ie, *x*, *y*, *z* planes) and angulation. This information is used to obtain a PSI guide to replicate the same implant positioning intraoperatively.

**Figure 2.**
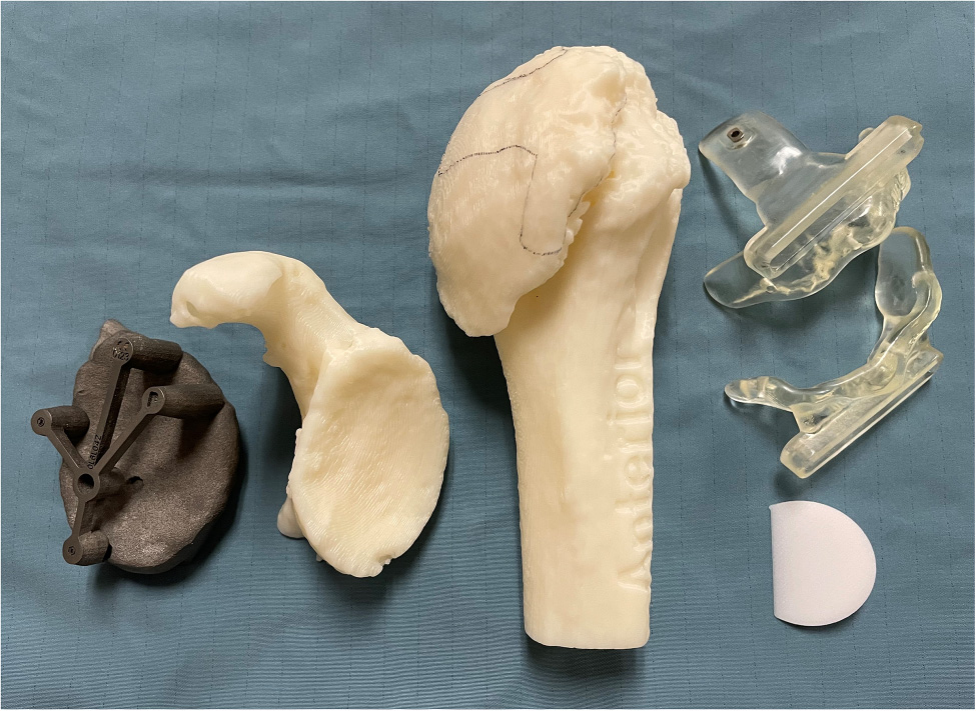
A PSI guide is obtained following three-dimensional (3D) templating. Two patient-specific cutting guides, the patient's scapula and humerus, and a replica of the bone graft with the same dimensions as the preoperative plan are 3D printed. All equipment is sterilized and used intraoperatively.

Once the dimensions of the patient-specific bone graft were defined as part of the preoperative plan, single-use patient-specific 3D-printed cutting guides are constructed at the Institute for Reconstructive Sciences in Medicine (iRSM, Edmonton), an internationally recognized center focusing on reconstructive medicine and the application of advanced digital technology in surgery. Two patient-specific cutting guides are developed in order to create sequential bone cuts of the proximal humerus. Additionally, the patient's scapula and humerus and a replica of the bone graft with the same dimensions as the preoperative plan are 3D printed (Figure 2). The 2 guides are placed on the 3D-printed humerus to assess for fit and correct placement. The 3D-printed graft is applied to the scapular template to ensure adequate correction of version and inclination and to determine the orientation of the graft on the glenoid face. All equipment is then sterilized in preparation for intraoperative use.

### Design of 3D-Printed Cutting Guides

**Figure 3.**
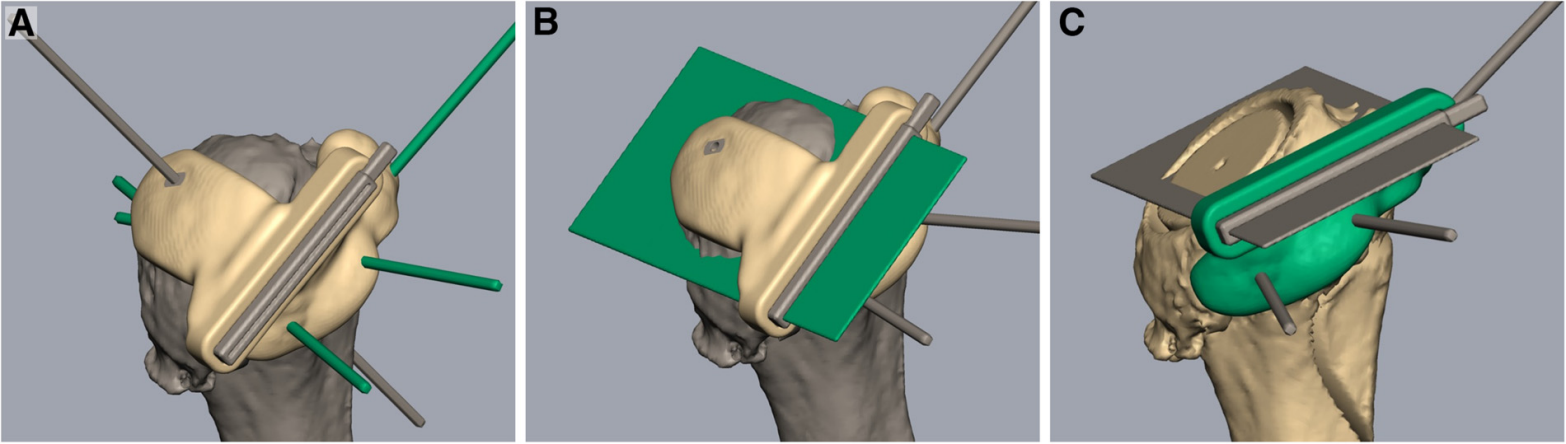
Design of the 3D-printed cutting guides. (A & B) A cutting plane is positioned to create a flat surface at the appropriate depth. (C) The second plane is positioned to create the angle of the graft required to address the glenoid deformity.

The patient's DICOM data are imported into Mimics Medical Version 21.0.0.406 software (Materialise NV) to create a 3D model of the patient's scapula and humerus. The graft dimensions are taken from the BLUEPRINT plan and used to create a 3D model using Rhinoceros 3D Version 7 SR16 software (Robert McNeel & Associates). A cylinder is created using the planned diameter of the graft. The minimum and maximum heights and angle are used to determine the cutting plane to create the graft. The cylinder and graft model are taken into Geomagic Freeform Version V2021.1.25 software (3D Systems, Inc) and positioned within the 3D model of the humeral head. The position of the graft model within the humeral head can be manipulated as necessary to avoid any areas with poor bone quality or cystic changes seen on preoperative CT. A cutting plane is positioned to create the cut that will provide the depth position and to create a flat surface (Figure 3A and B). The second plane is positioned to create the angle of the graft (Figure 3C). The 2 patient-specific cutting guides are designed around these planes. Digital files of the titanium cutting guide inserts and pilot hole drill guide were integrated into the design of the guides. Pins were positioned on the guide to optimize stability while the bone cuts are being performed. All planning and guide design was reviewed and verified with the surgical team and then prepared for 3D printing. The cutting guides were 3D-printed on the Form 3B additive manufacturing device at iRSM and printed in a biocompatible material that was validated for sterilization and approved by the hospital MDRD. The patient-specific anatomical models were printed on the F170 in ABS and on the Fortus 400mc in PC-ISO (polycarbonate material), also validated for sterilization. Once complete the models are postprocessed, verified for fit and sent to the requesting surgeon to be prepared for the operating room.

### Surgical Exposure, Graft Harvest, and Implant Insertion

**Figure 4.**
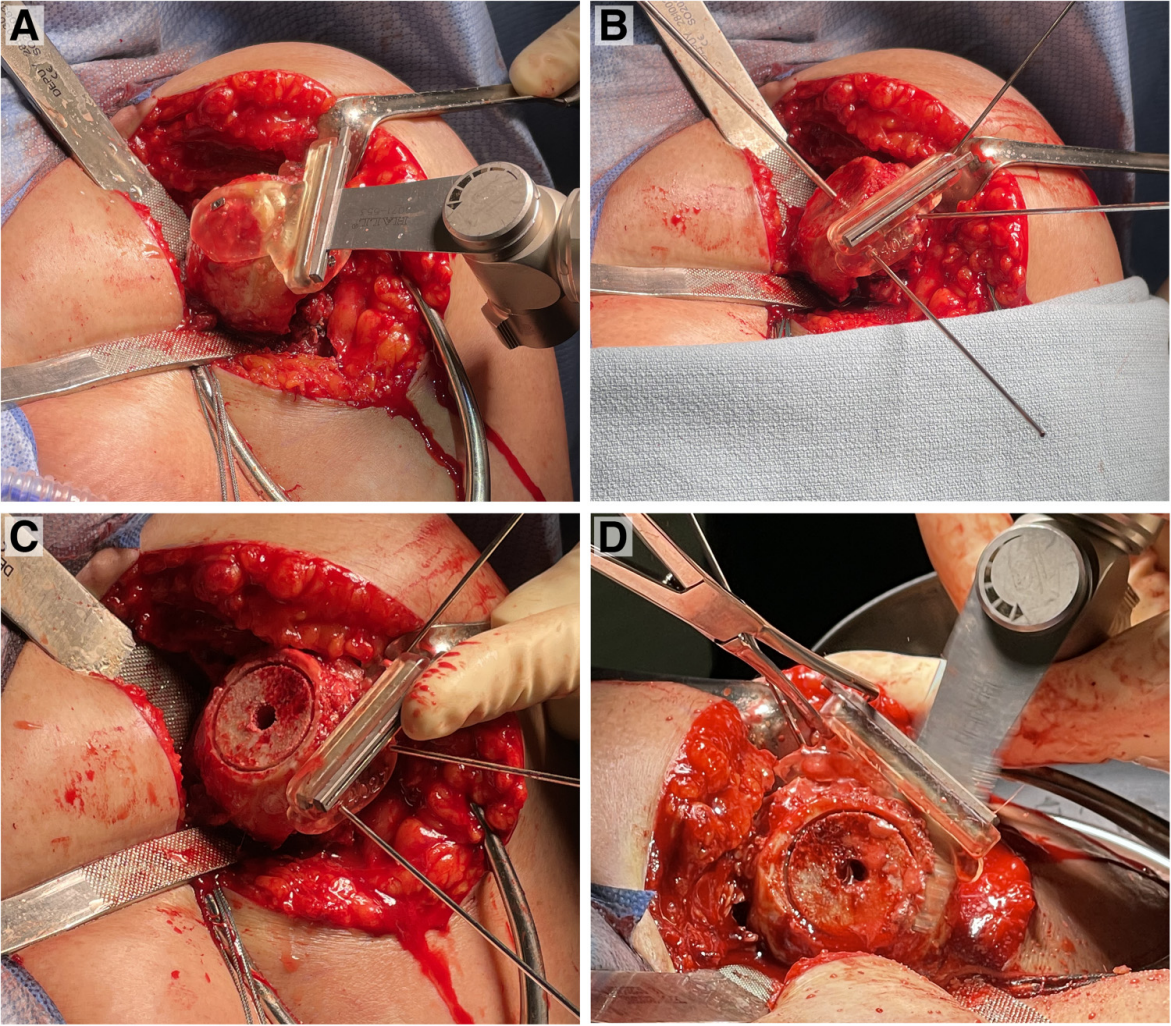
(A) The first three-dimensional (3D)-printed cutting guide is attached and secured to the proximal humerus with 3 smooth 3 mm pins and the first cut is made. (B) The first guide is removed and the centralization pin is reinserted into the proximal humerus. (C) After using a cannulated drill, a bell saw is passed over the central pin to create a circular-shaped dowel within the cancellous bone. (D) The second cutting guide is secured to the proximal humerus and used to cut the angled bone graft.

After regional (ie, interscalene nerve block) and general anesthesia, the patient is placed in the beach chair position with the head of bed at 30° to 40° of elevation. A standard deltopectoral approach is utilized. The biceps tendon, if present, is either tenotomized or tenodesed to the pectoralis major tendon. A subscapularis peel is performed, which includes careful capsular release along the humeral neck, to expose the glenohumeral joint. With gentle adduction, extension and external rotation of the shoulder, the shoulder is dislocated and the humeral head and proximal humerus exposure is optimized. Next, a series of stepwise bone cuts is performed utilizing 2 3D-printed cutting guides. Minimal osteophyte removal is performed to maintain the native anatomy of the proximal humerus. The first 3D-printed cutting guide is made to fit the exact contour of the proximal humerus, which is secured using 3 smooth 3 mm pins (2 parallel pins and 1 cross pin) (Figure 4A). An alignment pin is then drilled through the central hole of the guide in order to mark the center position of the eventual graft. The alignment pin is temporarily removed and an oscillating saw is then passed through the slotted metallic insert within the guide to remove the dense sclerotic subchondral bone of the humeral head and create a flat cut surface. The single cross pin securing the guide is then removed and the first guide is removed from the proximal humerus (Figure 4B). The central alignment pin is then reinserted into the proximal humerus and a cannulated drill that is similar in diameter to the central post of the glenoid baseplate is then passed over the central pin. Next, a 26 mm diameter bell saw is passed over the central pin to create a small circular-shaped dowel within the cancellous bone of the humeral metaphysis. The second 3D-printed guide is then secured to the proximal humerus, utilizing the previously inserted parallel k-wires and the off-axis wire (Figure 4 C and D). An oscillating saw is then used to cut an angled bone graft exactly matching the Blueprint™ plan. The graft is placed on the back table and compared to the 3D-printed graft template. A free-hand humeral cut is then performed along the anatomic neck at the desired humeral version, followed by preparation of the proximal humerus in standard fashion.

**Figure 5.**
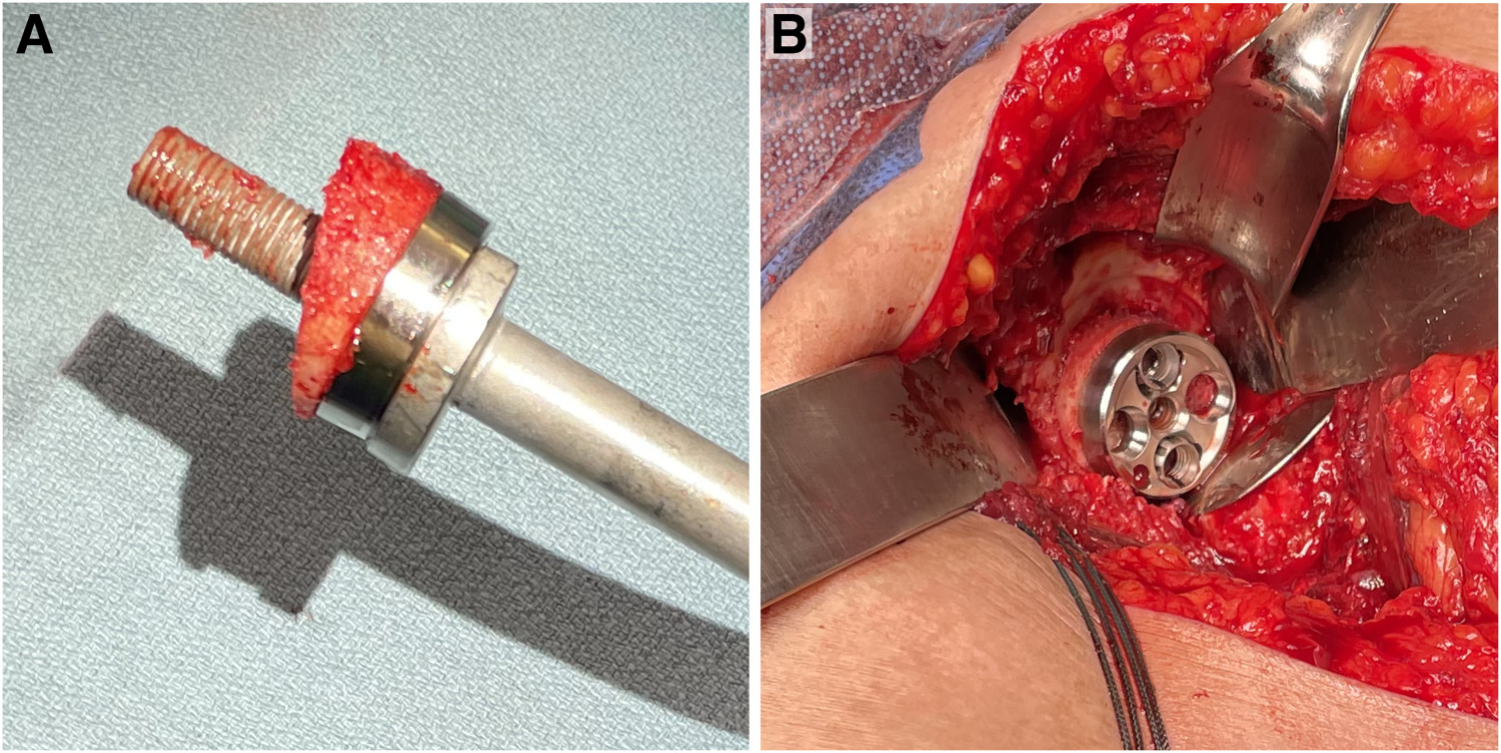
(A) The angled graft is inserted on the glenoid baseplate. (B) The baseplate is impacted onto the native glenoid with the graft oriented to match the point of maximal defect on the glenoid.

The glenoid is exposed and cleared of labrum and residual cartilage. The bony anatomy of the glenoid including rim osteophytes is preserved to ensure accurate positioning and seating of the PSI guide and placement of the glenoid guide pin (ie, replicating the preoperative plan). The glenoid surface is then thoroughly assessed and the point of the “maximal glenoid defect” is marked with electrocautery and compared to the 3D-printed glenoid and preoperative plan prior to high-side reaming.^22^ Care is taken during reaming to preserve the native glenoid deformity to allow proper seating of the harvested graft and thereby optimize baseplate stability. The native glenoid surface that does not undergo reaming is drilled with a 2 mm drill bit to create a bleeding bone surface, which has been demonstrated to aid in bone graft incorporation.^23^ The patient-specific bone graft is then placed over the central post of the glenoid baseplate, with the deepest portion of the graft properly oriented to match the previously marked “point of maximal defect,” and is then impacted until fully seated (Figure 5 A and B). While the cancellous bone graft is seated, it has the ability to compress and contour to any small residual undulations in the posterior/posterosuperior glenoid surface. The glenosphere is then inserted and secured. A trial reduction is performed prior to insertion of the definitive humeral component ensuring adequate soft-tissue tensioning, stability, and absence of perimeter impingement. The joint and surgical wound is then thoroughly irrigated and closed in layers in the standard fashion.

## Results

### Case

**Figure 6.**
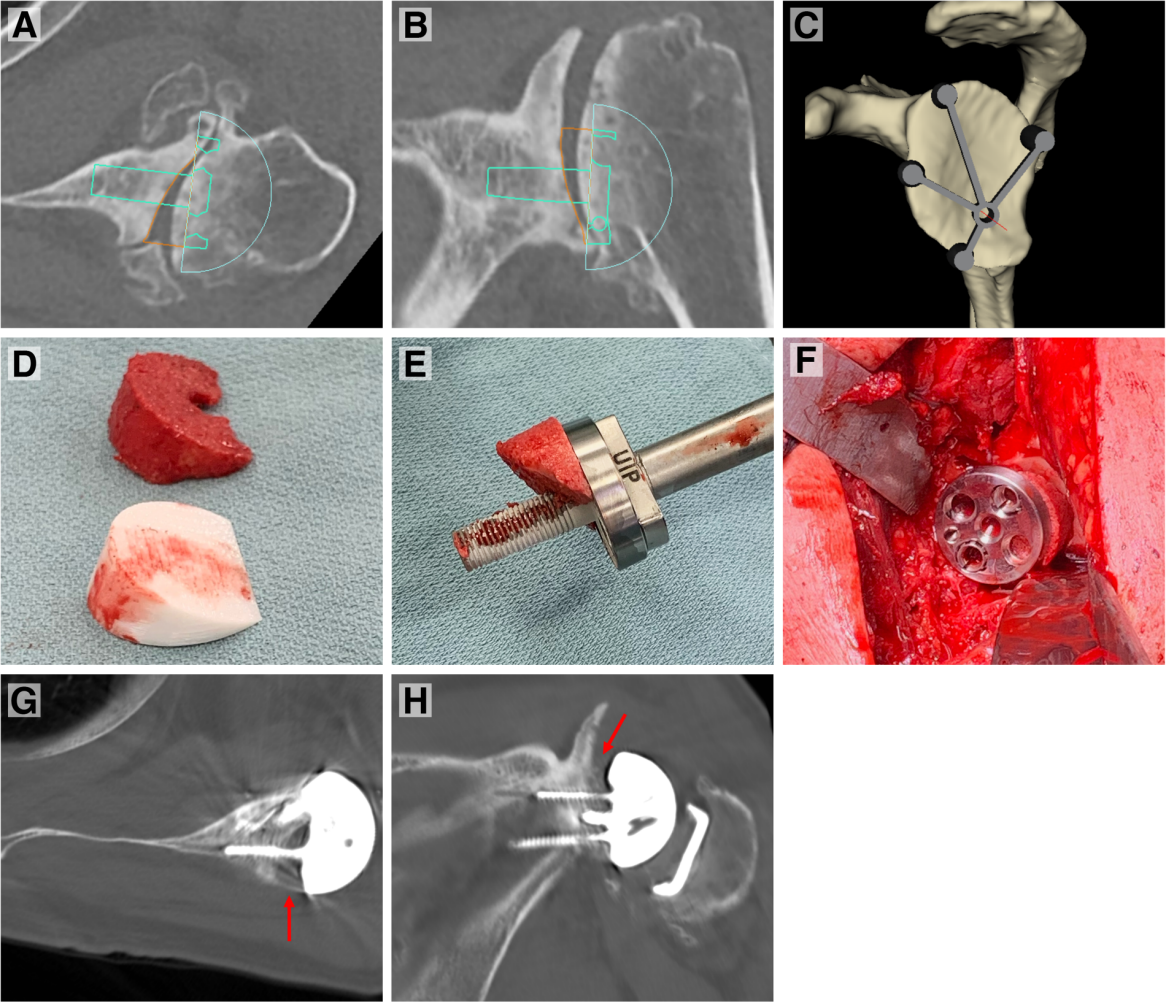
(A, B, and C) Preoperative 3D templating of the presented case revealing substantial glenoid erosion and deformity (ie, retroversion). Glenoid baseplate positioning, graft dimensions, and central pin placement is templated. (D) Comparison of graft harvested utilizing the 3D-printed cutting guide and the 3D-printed replica of the wedge deemed necessary to correct the patient's deformity. (E and D) Intraoperative pictures showing the graft inserted on the glenoid baseplate and impacted onto the glenoid in appropriate orientation. (G and H) Postoperative axial and coronal CT images demonstrating correction of glenoid version and inclination and position of the bone graft. (Arrows pointing to the bone graft positioned under the glenoid baseplate). Abbreviations: 3D, three-dimensional; CT, computed tomography.

A 69-year-old, right-hand dominant electrician presented with left shoulder pain exacerbated by repetitive work and weakness resulting from end-stage osteoarthritis. He received a contralateral right RSA with bone autograft utilizing a free-hand technique 1 year prior due to ongoing pain and dysfunction. On examination of the left shoulder, there was 90° of active forward flexion, neutral external rotation and internal rotation to the lateral hip. Preoperative planning using 3D CT software revealed 33° of glenoid retroversion and 3° of superior inclination. Our goal for correction determined by the PSI guide was 7° of retroversion and 3° of inferior inclination. The dimensions of the bone graft to achieve this correction were calculated to be 29 mm in diameter, 12 mm in height, with an angle of 28.4°. While performing the preoperative design of the cutting guide, multiple cysts in the humeral head were noted. The initial planned resection planes would have resulted in a wedge of bone through this area of poor-quality bone. Utilizing the planning software, the planned wedge of bone was rotated and translated medially in order to avoid this cystic area. The patient underwent an RSA with bone grafting utilizing the above technique with 3D-printed cutting guides without complication. Preoperative and postoperative radiographs and CT are demonstrated in Figure 6. Baseplate version was measured postoperatively using a CT scan via the Friedman method,^24^ showing 7° of retroversion. Inclination was found to be 2° of inferior inclination using the beta angle method.^25^ At his 6-week follow-up, the patient was doing excellent with minimal pain. On examination, he was able to achieve 140° of forward flexion and approximately 5° of external rotation.

## Discussion

Acquired glenoid bone loss is found in up to 39% of patients undergoing an RSA with 15% requiring bone grafting.^9^ Substantial glenoid bone loss in the setting of primary or revision arthroplasty has been associated with inferior outcomes,^7^ raising several potential issues including component instability, mechanical impingement and scapular notching, poor soft-tissue tensioning and limited options for revision procedures. Glenoid defect patterns are determined based on their etiology. Glenohumeral arthritis is associated with posterior wear,^26^ rotator cuff arthropathy with posterosuperior wear,^27^ chronic anterior instability with anterior wear^28^ and revision cases with universal bone loss.^29^ Walch et al^26^ described the most commonly used classification system for glenoid erosion, as follows: A1, concentric; A2, concentric and centrally eroded; B1, posteriorly subluxated; B2, posteriorly subluxated and eroded; and C, retroverted and hypoplastic. Sirveaux et al^30^ expanded upon this and created a classification system to describe superior glenoid wear in patients with CTA: Grade E0, no wear; E1, central concentric wear; E2 and E3, superior wear; and E4, inferior glenoid erosion.

In order to address glenoid erosion, multiple techniques have been described including eccentric reaming, trabecular metal augmented glenoid baseplates, and bone grafting.^9,17,31,32,33^ At our institution, utilizing an angled bone graft harvested from the humeral head (ie, autograft) is the preferred technique as it offers a biologic solution with the advantages of addressing the glenoid defect, reconstituting the joint line and increasing the available bone stock for future revision scenarios.^34^ A recent systematic review by Malahias et al^35^ including 13 studies (539 shoulders) on glenoid bone grafting in RSA concluded that bone grafting the glenoid with autograft or allograft bone is associated with satisfactory clinical function and radiographic outcomes in the short-term with a low rate of complications requiring surgery (3.5%). In the primary setting, rate of radiographic graft nonunion was 5.2%, glenoid component migration was 0.4% and infection rate was 0.9%. Additionally, Lanham et al^36^ published a systematic review including 19 studies (652 shoulders) comparing bone grafting and augmented glenoid baseplates and found that both groups had similar clinical outcomes, complications and revision rates; however, there was a slightly higher rate of infection and scapular notching in the bone grafting group. An increased rate of infection when performing bone grafting has been postulated to be due to the increased surgical time that is required to create the bone graft, as increased surgical time has been shown to increase the rate of prosthetic joint infections in hip and knee arthroplasty.^37^ Although utilizing patient-specific cutting guides increases the time needed for preoperative planning, we hypothesize the intraoperative time required to create such grafts will be decreased. More data is required, however, to test this hypothesis.

Preoperative planning with 3D planning software is a crucial step in this technique to ensure an accurate assessment of the glenoid bone loss and bone graft required to address the deformity and positioning of the glenoid baseplate.^38,39,40^ Our goal is to implant the baseplate within 10° of neutral version and full correction of glenoid inclination (ie, at least to 0°). Using these parameters during the preoperative planning stage, we can determine the exact dimensions of a graft required for each patient (ie, patient-specific graft). Multiple studies have assessed the accuracy of glenoid baseplate implantation utilizing 3D planning software. Iannotti et al^41^ determined that the use of standardized instrumentation and 3D planning improved guide pin positioning compared to standard instruments and two-dimensional planning in anatomic shoulder arthroplasty. This finding was replicated in patients requiring RSA and bone grafting.^42^ In contrast, whether glenoid PSI guides are necessary to improve the positioning of the glenoid baseplate in cases with glenoid deformity has more conflicting results. Eraly et al^43^ created glenoid defects in 10 cadaveric shoulders and implanted an RSA using patient-specific guides versus standard instrumentation. The patient-specific guides revealed significant reductions in angular deviation from the planned glenoid implant position. In addition, the use of the intraoperative guide permitted surgeons to obtain 89% of the preoperatively planned intraosseous screw length compared with 52% in the group without a guide. Tashjian^42^ compared baseplate positioning in 15 patients requiring a bone graft using 3D planning and standard instrumentation versus 3D planning and a PSI guide. Although a significant difference in final retroversion of 6° was found, this value was not deemed clinically meaningful and recommended that PSI guides may be more useful to address severe deformities (ie, B3 or E3 glenoid erosion). Although there may be conflicting results in regard to the necessity of PSI guides in shoulder arthroplasty, our experience has been favorable and therefore we recommend their use as an integral step in this surgical technique to ensure proper base plate positioning and subsequently correct positioning of the bone graft on the prepared glenoid in order to restore glenoid alignment.

The medical applications of 3D printing are rapidly evolving. 3D-printed guides have been extensively used in maxillary and particularly mandibular reconstructive surgery to replace what was previously considered a gold standard in treatment using fibular osseous vascular free flaps.^44,45,46,47^ Procedures that utilize these guides such as in mandibular reconstructive surgery have demonstrated reduced surgical time and overall improvements in the accuracy, safety, and reliability of these procedures.^47,15^ In the field of orthopedic surgery, patient-specific cutting guides have been used for the distal femur, proximal tibia,^13^ pelvis^14^ and the midfoot,^15^ and have demonstrated excellent surgical accuracy, ease of use, and superior results over free-hand techniques. Additionally, a study by Jaffry et al^16^ demonstrated that the use of 3D-printed instruments for unicompartmental knee arthroplasty revealed a good correlation with implants placed by robot-assisted surgery.

To our knowledge, the use of 3D-printed cutting guides has not been described in shoulder arthroplasty. Although the outcomes have not yet been delineated, we see many potential advantages to this technique. The cutting guides allow for precise replication of the bone graft that was templated preoperatively using 3D planning software to ensure accurate correction of the glenoid deformity and appropriate glenoid baseplate positioning. Preoperatively, we are able to modify which area the cutting guide harvests the wedge of bone from on the humeral head in order to avoid areas of cystic changes. Furthermore, by presenting a standardized technique to create a bone graft, we anticipate that the overall surgical time for this step of the procedure will be decreased, which in turn, may decrease postoperative infection rates. However, short- and long-term studies are required to verify these potential advantages.

## Limitations

Potential limitations of this novel technique include the necessary access to an institution that has the ability to 3D print and manufacture the cutting guides required to create the bone graft, thereby limiting the generalizability of this technique to other surgical centers. The preoperative planning phase of this technique takes longer than when using standard instrumentation due to the time required to obtain advanced imaging and a PSI guide, and the time needed to create the 3D-printed guides. Additionally, a specific limitation in our healthcare system is the time lag between acquiring the initial preoperative CT and the surgical date. If this time is lengthy (ie, greater than 6-12 months), the patient's anatomy may subtly change with the progression of arthritis, which may compromise the exact fit of the cutting guides on the proximal humerus in addition to the dimensions of the bone graft that is required to address the glenoid deformity. Finally, it is yet to be determined whether this technique will be accurate and clinically relevant in the short- and long-term compared to standard instrumentation. In the case example presented here, only short-term results are available; long-term clinical and radiographic outcome data is required to determine the efficacy of this technique.

## Conclusion

The authors have described a novel technique using 3D-printed cutting guides to create a patient-specific bone graft to address severe glenoid deformity in the setting of RSA.
